# Performing the Millikan experiment at the molecular scale: Determination of atomic Millikan-Thomson charges by computationally measuring atomic forces

**DOI:** 10.1063/1.5001254

**Published:** 2017-09-07

**Authors:** T. Ryan Rogers, Feng Wang

**Affiliations:** Department of Chemistry and Biochemistry, University of Arkansas, Fayetteville, Arkansas 72701, USA

## Abstract

An atomic version of the Millikan oil drop experiment is performed computationally. It is shown that for planar molecules, the atomic version of the Millikan experiment can be used to define an atomic partial charge that is free from charge flow contributions. We refer to this charge as the Millikan-Thomson (MT) charge. Since the MT charge is directly proportional to the atomic forces under a uniform electric field, it is the most relevant charge for force field developments. The MT charge shows good stability with respect to different choices of the basis set. In addition, the MT charge can be easily calculated even at post-Hartree-Fock levels of theory. With the MT charge, it is shown that for a planar water dimer, the charge transfer from the proton acceptor to the proton donor is about −0.052 *e.* While both planar hydrated cations and anions show signs of charge transfer, anions show a much more significant charge transfer to the hydration water than the corresponding cations. It might be important to explicitly model the ion charge transfer to water in a force field at least for the anions.

## I. INTRODUCTION

Electrons show marked quantum behavior and thus are always delocalized in space. Their delocalized distribution along with the fact that electrons are indistinguishable particles with Fermion symmetry makes unique assignments of electrons to atoms in a molecule challenging if not impossible. On the other hand, partial charge assignments, or population analysis, provide valuable insights into the nature of intermolecular interactions. In the area of force field development, atomic partial charges directly influence the forces experienced by each atom and play a crucial role in determining the Coulombic interaction, which is one of the most important components in the energy of molecular systems.

Given the challenges in partial charge determination, it is not surprising that a large variety of methods have been reported, such as Mulliken analysis,^1^ Löwdin partitioning,^2,3^ distributed multipole analysis,^4^ Hirshfeld charge analysis,^5^ Bader’s atoms in molecules (AIM),^6^ and methods based on absolutely localized orbitals,^7^ or Ruedenberg^8^ and Knizia^9^ orbitals.^10^ All of these methods have their strengths and weaknesses. Although some of these methods are widely implemented, especially the simpler ones, some more advanced methods only acquired limited support in popular computational packages.

Assuming the charge distribution in a molecule can indeed be represented with atomic charges, dipoles, and higher order moments up to infinite order, it can be shown that the potential energy, *U*, in an external electric field is

$$\begin{array}{ccc} U & = & \underset{i}{\sum }M_{i}V_{i}+\underset{i}{\sum }\underset{\alpha }{\sum }M_{i,\alpha }V_{i,\alpha }\\ & & +\;\frac{1}{2}\underset{i}{\sum }\underset{\alpha ,\beta }{\sum }M_{\alpha \beta }{V_{i,}}_{\alpha \beta }+\cdots , \end{array}$$ (1)

where the sum is over the atom index *i* and the Cartesian directional indices *α* and *β. M* is the zeroth moment, which is the partial charge; *M*_*α*_ is the first moment or the dipole moment; *M*_*αβ*_ is the second moment, which can be replaced by the traceless quadrupole moment without affecting the validity of Eq. (1). $V_{i}$ would be the external potential at atom *i*, with ${V_{i,}}_{\alpha }=\partial V_{i}/\partial V_{i}\partial \alpha \partial \alpha$ being the first derivative of the potential, which is the negative of the electric field. *V*_*i*,*αβ*_ are thus the negatives of the field gradients. It should also be noted that the dipole and quadrupole moments being used in Eq. (1) should include both the permanent and induced moments in the external field.

In a uniform electric field, Eq. (1) simplifies since the field gradient and higher order terms will be zero. For simplicity, if we assume the uniform field is along the direction *γ*, Eq. (1) reduces to

$$U=\underset{i}{\sum }M_{i}V_{i}+\underset{i}{\sum }M_{i,\gamma }V_{i,\gamma },$$ (2)

where *i* is the atomic index.

Now, consider the force on atom *i* along the direction *γ*, $F_{i,\gamma }=-\frac{\partial U}{\partial \gamma_{i}}$ in the presence of the uniform field. When taking this derivative following the chain rule, all terms involving field gradients will be zero since the field is uniform. If one ignores the charge flow terms $\frac{\partial M_{i}}{\partial \gamma_{i}}$ and $\frac{\partial M_{i,\gamma }}{\partial \gamma_{i}}$, the atomic forces will become

$$F_{i,\gamma }=-M_{i}V_{i,\gamma }=-M_{i}V_{\gamma }.$$ (3)

The subscript of the field on atom index is dropped since the field is uniform everywhere. Thus, if the charge flow terms can be ignored, the atomic forces will only be along the direction of the field.

Unfortunately, in general, the charge flow terms cannot be ignored; however, it can be shown that the charge flow terms are exactly zero for planar molecules when the force is measured in a direction perpendicular to the molecular plane, making Eq. (3) exact.

It is important to note that the charge flow terms describe the response of a charge distribution to the motion of atomic coordinates rather than the response of the charge distribution to an external field. The latter is generally referred to as induction. An induced dipole moment does not contribute to atomic or molecular forces when the field gradient is zero. We also note that we use the term “charge flow” to include electron redistribution within an atomic volume, and the term “charge transfer” usually implies the transfer of electron density from one atomic volume to another.

For any molecule, due to the translational and rotational invariance, the charge distribution, *ρ*, can be uniquely determined by 3N-*m* independent interatomic distances, where *m* accounts for the overall translational and rotational degrees of freedom. This is true since the full Schrödinger’s equation of the system can be written in terms of internal coordinates of the nuclei and there are only 3N-*m* independent variables to uniquely define the system.

The charge flow due to perturbation of the coordinate of atom *i* along *α* can be calculated as

$$\frac{\partial \rho \left(\tilde{r}\right)}{\partial \alpha_{i}}=\underset{j}{\sum }\frac{\partial \rho }{\partial r_{j}}\cdot \frac{\partial r_{j}}{\partial \alpha_{i}},$$ (4)

where $\tilde{r}$ stands for the set of independent interatomic distances, each member of the set is labeled with *j* in the sum. *ρ* is the overall charge distribution from solving the quantum mechanical, many-electron Hamiltonian. All orders of the multipole moment, *M,* can be determined from the knowledge of *ρ*.

We consider a planar molecule in the x-y plane with an infinitesimal motion of atom *i* along the z direction. For any distance *r*_*j*_ between two arbitrary atoms A and *B*,

$$\frac{\partial r_{AB}}{\partial z_{i}}=\begin{array}{ccc} 0 & & A,B\neq i,\\ \frac{z_{i}-z_{B}}{r_{iB}} & & A=i,\\ \frac{z_{i}-z_{A}}{r_{Ai}} & & B=i. \end{array}$$ (5)

The derivatives in Eq. (5) are always zero since all atoms in a planar molecule have the same *z* coordinate. For planar molecules, Eq. (4) thus indicates that the electron distribution remains constant when an infinitesimal displacement along *z* is made, regardless whether there is an external field or not. In other words, there would be no charge flow with respect to any infinitesimal displacement of any atom normal to the plane. This no-charge-flow condition leads to Eq. (3) being exact for planar molecules in a uniform field.

We note that a relationship similar to Eq. (3) is the basis for the determination of the charge of an electron in 1909 by Robert A. Millikan’s experiment,^11,12^ where the gravitational force is balanced by the Coulombic force of a charged drop of oil in an external field. Equation (3) is an atomic version of the Millikan experiment; although it is worth emphasizing that Eq. (3) only holds under the no-charge-flow condition, which can only be rigorously satisfied for a planar molecule. If we use the symbol *q*_*i*_ as the atomic charge, Eq. (3) can be rewritten as

$$q_{i}=\frac{F_{i,z}}{E_{z}},$$ (6)

where the potential derivative *V*_*z*_ is replaced with the more familiar symbol for the electric field, $E_{z}$.

The charge measured following such a procedure will be referred to as the Millikan-Thomson (MT) atomic charge in honor of Robert A. Millikan and Joseph J. Thomson. Both relied on force experienced by a charged particle in a uniform electric field to determine fundamental properties of the electron.^12,13^ Although it would have been sufficient to refer to this charge as the Millikan partial charge, there is a good chance of confusion with the Mulliken charge proposed by Robert S. Mulliken.^1^ Technically, an experiment can be designed to measure the atomic MT charges. In this work, we will restrict ourselves to theoretical determination of the MT charges.

We note that for planar molecules, an intuitive way to understand the no-charge-flow condition is to consider the symmetry of the molecule. If the direction of the field is reversed, the MT charge cannot change due to symmetry. If the charge flow has a contribution to the MT charge to the first order of the field, the contribution must change sign when the field reverses direction. This will lead to the conclusion that the charge follow term cannot exist for a planar molecule to the first order. However, care must be taken when applying this argument since the existence of a symmetry plane is not sufficient to guarantee no charge flow. Consider a water molecule, where two symmetry planes exist. One would get the correct MT charge only if the field is applied perpendicular to the water plane. If the field is applied perpendicular to the other symmetry plane, containing only the oxygen atom, the electrons will redistribute along the molecule. Such a redistribution is strongly coupled to all the atomic coordinates and will affect not just the hydrogen atoms. The equilibrium geometry of water in such a field will change leading to the two OH bonds being non-equivalent. Thus if one attempts to measure the MT charge by applying a field perpendicular to the symmetry plane that mirrors one hydrogen to the other, a different charge will be obtained. Even for the oxygen atom that is in the symmetry plane, the charge will be affected by the charge flow contribution. This contribution will reduce the apparent MT charge by more than 20% according our numerical validations.

It is important to mention that, for strictly planar molecules, the MT charge is actually the same as the IR charge derived previously by Milani and Castiglioni.^14^ The IR charge was calculated by evaluating the dipole derivative with respect to atomic coordinates relative to a local plane.^15^ A local plane was chosen for the purpose of minimizing charge flow. The Milani and Castiglioni work also showed that the charge flow term is exactly zero for planar molecules.

The MT charge is a double derivative of the total quantum mechanical potential energy with respect to the external field and the atomic coordinate along the direction of the field,

$$q_{i}=-\frac{\partial^{2}U}{\partial E_{z}\partial z_{i}}.$$ (7)

If the field derivative is taken before the coordinate derivative, the MT charge can be understood as the dipole moment derivative with respect to atomic coordinates. Thus the MT charge is the same as the IR charge. Since the IR charge can be defined for non-planar molecules using a local plane, the MT charge can also be measured similarly relative to a local plane. However, in such cases, the charge flow contribution is minimized but not completely eliminated. In this paper, we will restrict ourselves mostly to planar molecules, which are completely free of charge flow contributions.

Another similar definition is the Generalized Atomic Polar Tensor (GAPT) charge, which was defined by using the full trace of the dipole derivate tensor.^16,17^ For GAPT charges, the contribution from charge flow will not be zero even for planar molecules.

Although it could be argued that the MT charge is simply a new perspective for the IR charge, the MT charge determines the atomic force experienced by an atom under an external field. Thus it is arguably the most relevant charge to use when a model potential is developed. We note this argument is not restricted to the development of a point charge potential. Eq. (1), on which the derivations are based, is completely general. A polarizable potential with distributed atomic multipoles should also reproduce the forces felt by the MT charges in a uniform field.

It is worth mentioning that the MT charge will necessarily reproduce the molecular dipole moment since the MT charges can be calculated by taking the molecular dipole derivative with respect to the out-of-plane atomic displacements as done with the calculations of IR charges. Since no atomic distance of a planar molecule will change by an infinitesimal displacement in the out-of-plane direction, the out-of-plane derivative of the molecular dipole is thus similar to a rotation of the dipole. In this case, the derivative will be the dipole itself. This is clearly shown in Table II (*vide infra*).

It has been argued that the determination of the IR charge is costly due to the need to obtain the dipole derivative tensor and the IR spectra.^15,18^ The equivalence shown in this work allows this charge to be determined by a single force calculation performed under an external field. Since only forces along the field direction are required, a numerical differentiation of the energy will not be very costly. Each charge only requires two energy evaluations, opening up the possibility of obtaining partial charges with highly correlated levels of theory, such as CCSD(T). We also want to emphasize that the MT charges can be determined with any existing electronic structure code as long as the code supports evaluating energies under an external field. This is a huge convenience in terms of implementation compared with many other population analysis methods.

In Sec. II, the MT partial charges of selected systems will be reported. Comparison with some existing population analysis methods will be performed for some of these molecules. Since the MT charge could arguably be measured experimentally, we think it provides a good reference for other population analysis methods. The MT charges for the water dimer and several hydrated ions are also studied to provide some insight into the importance of charge transfer in liquid water and ionic solutions. A summary and conclusion will be provided in Sec. III.

## II. DETERMINATION OF MT ATOMIC CHARGES

**FIG. 1.**
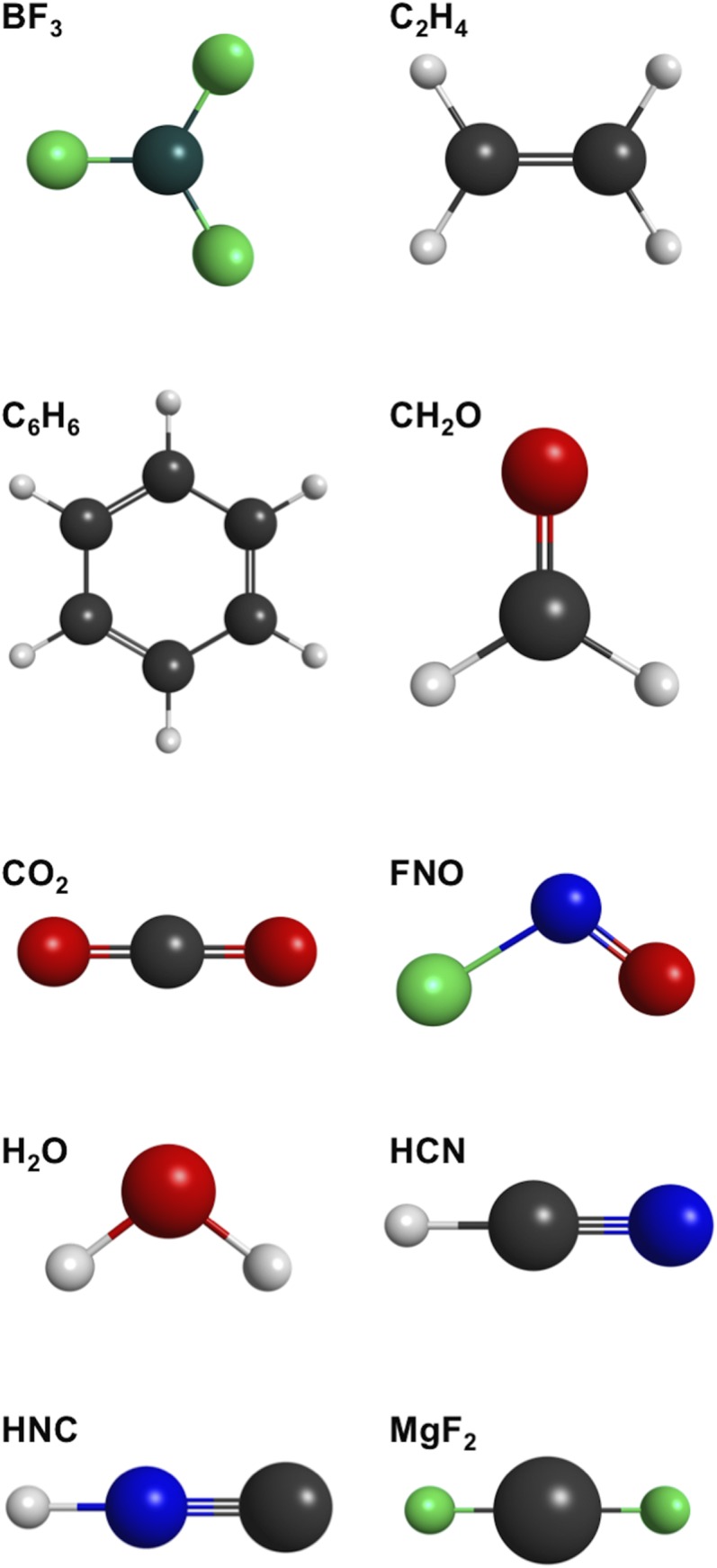
Ball-and-stick models of all molecules listed in Table I. Molecular formulae are given at the top left of each structure.

Table I reports the MT charge for BF_3_, C_2_H_4_, C_6_H_6_, CH_2_O, CO_2_, FNO, H_2_O, HCN, HNC, and MgF_2_, calculated at B3LYP, MP2, and CCSD(T) levels of theory. Only symmetry unique atoms are listed in Table I and the corresponding structures are shown in Fig. 1. All the calculations were performed with a tight HF density convergence of 1 × 10^−8^. The MT charge determinations were performed with three different basis sets:^19–21^ aug-cc-pVDZ, aug-cc-pVTZ, and aug-cc-pVQZ for B3LYP and MP2. Only the aug-cc-pVTZ basis set was used for the CCSD(T) determinations. All the calculations were preformed with the GAMESS suite of programs.^22^ Whereas analytical gradients can be used for MP2 calculations, both B3LYP and CCSD(T) charges were determined using finite difference of energies to obtain with the out-of-plane force. The external field used for the calculations is 0.001 atomic units, and the finite difference in energy is performed with a displacement of 0.001 Å in both the positive and negative z directions. The formula is thus accurate up to the third order of the displacement.

**TABLE I.** Millikan-Thomson charges calculated for selected molecules (Fig. 1). Only the MT charges on the symmetry-unique atoms are shown. AVXZ stands for the aug-cc-pVXZ basis set. All charges are reported in the elementary charge unit, *e*. B3LYP computations were performed on geometries optimized under B3LYP and aug-cc-pVDZ; both MP2 and CCSD(T) computations were performed on geometries optimized under MP2 and aug-cc-pVDZ.

		B3LYP			MP2			
Molecule	Atom	AVDZ	AVTZ	AVQZ	AVDZ	AVTZ	AVQZ	CCSD(T) AVTZ
BF_3_	B	0.923	0.953	0.956	0.956	0.979	0.985	0.985
	F	−0.308	−0.318	−0.319	−0.319	−0.326	−0.328	−0.328
C_2_H_4_	C	−0.297	−0.293	−0.292	−0.290	−0.284	−0.283	−0.279
	H	0.148	0.147	0.146	0.145	0.142	0.142	0.140
C_6_H_6_	C	−0.134	−0.133	−0.132	−0.136	−0.133	−0.133	−0.131
	H	0.134	0.132	0.132	0.136	0.133	0.133	0.131
CH_2_O	C	0.137	0.138	0.138	0.126	0.121	0.123	0.123
	H	0.094	0.093	0.093	0.096	0.099	0.099	0.098
	O	−0.324	−0.324	−0.324	−0.318	−0.318	−0.320	−0.320
CO_2_	C	0.507	0.516	0.514	0.438	0.441	0.443	0.483
	O	−0.253	−0.258	−0.257	−0.219	−0.220	−0.221	−0.242
FNO	F	−0.215	−0.213	−0.213	−0.291	−0.284	−0.284	−0.263
	N	0.142	0.142	0.141	0.163	0.158	0.159	0.162
	O	0.072	0.072	0.072	0.128	0.126	0.125	0.102
H_2_O	O	−0.655	−0.655	−0.655	−0.657	−0.652	−0.654	−0.647
	H	0.327	0.327	0.327	0.329	0.326	0.327	0.324
HCN	H	0.260	0.259	0.259	0.259	0.257	0.256	0.257
	C	0.047	0.050	0.050	0.037	0.042	0.044	0.039
	N	−0.307	−0.309	−0.308	−0.297	−0.299	−0.300	−0.296
HNC	H	0.414	0.408	0.408	0.413	0.406	0.405	0.407
	N	−0.230	−0.220	−0.220	−0.193	−0.183	−0.184	−0.218
	C	−0.185	−0.188	−0.188	−0.220	−0.222	−0.222	−0.189
MgF_2_	Mg	1.417	1.408	1.408	1.466	1.458	1.456	1.462
	F	−0.709	−0.704	−0.704	−0.733	−0.729	−0.728	−0.731

One would assume that analytical gradient calculations can be performed at least for the B3LYP MT charge calculations. It surprised us that some electronic structure programs fail to give consistent analytical gradients for planar molecules in the presence of an external field. For example, the total force for an H_3_O^+^ cation should be the charge *e* times the field *E*_*z*_. If the atomic forces do not sum to the correct total force, we will consider the result inconsistent. These likely reflect bugs in the implementation of the analytical gradient routine in the presence of the field. Due to the good agreement between the numerical gradients evaluated using our finite difference procedure and the analytical gradients in cases where the analytical gradients are known to be correct, we will resort to using numerical gradients when we are not confident that the analytical gradients are consistent.^23^

Before any numbers are reported in this work, we ensure that all symmetry unique atoms must have identical atomic forces. In addition, the sum of out-of-plane forces must give a total force of *q*_total_·*E*_*z*_, where *q*_total_ is the total charge of the molecule. We will refer to this total force constraint as the Millikan constraint since it has to hold for the historical Millikan experiment to be valid.

For all the molecules in Table I, the geometries were optimized at the MP2 level of theory with the aug-cc-pVDZ basis set for all the MP2 and CCSD(T) computations, while geometries for the B3LYP computations were optimized by B3LYP with the aug-cc-pVDZ basis set. It is clear that the MT charges have very weak dependence on the basis set. The MP2 charges in general are giving better agreement with the CCSD(T) charges than B3LYP with the exception of CO_2_. It is interesting that CO_2_ seems to pose the most challenge to MP2 with MP2 underestimating the CCSD(T) MT charges by around 10%. The agreement between B3LYP and CCSD(T) is actually slightly better than that for MP2.

For most molecules, the B3LYP MT charges are in good agreement with the CCSD(T) references. The largest error is in the case of FNO, where B3LYP underestimates the MT charges on F and O by around 30% when compared to the CCSD(T) reference. The error for MgF_2_ is also fairly large, with B3LYP underestimating the MT charges by around 5%. The DFT description thus underestimates the ionic character of the bond. The same underestimation is also present for BF_3_ except to a smaller extent. These disagreements indicate a problem with the DFT description of the charge distribution for these molecules.

Table II summarizes the dipole moments of all the polar molecules calculated by GAMESS and from the MT charges. For BF_3_, C_2_H_4_, C_6_H_6,_ CO_2_, and MgF_2_, the total molecular dipole moments are zero due to symmetry and are thus not included in Table II. For B3LYP and MP2, the dipole moments were determined with the aug-cc-pVQZ basis set. For CCSD(T), the corresponding basis set is aug-cc-pVTZ. While GAMESS supports analytical dipole moment for B3LYP and MP2, the CCSD(T) dipole moment can only be obtained numerically by finite difference with respect to an external field. Thus for CCSD(T), the dipole moments were only calculated from the MT charges. As discussed previously, the MT charge will necessarily reproduce the molecular dipole moments. This is clearly true by our numerical verification with B3LYP and MP2. Except for FNO, for all the molecules in Table II, the B3LYP dipole is in close agreement with MP2 and CCSD(T) dipole moments.

**TABLE II.** Dipole moments calculated in GAMESS and compared to those derived from MT charges. The dipole moments are reported in units of Debye. AVXZ stands for the aug-cc-pVXZ basis set. B3LYP computations were performed on geometries optimized under B3LYP and aug-cc-pVDZ; both MP2 and CCSD(T) computations were performed on geometries optimized with MP2 and aug-cc-pVDZ.

	B3LYP AVQZ		MP2 AVQZ		
Molecule	GAMESS	MT	GAMESS	MT	CCSD(T) AVTZ MT
CH_2_O	2.406	2.408	2.433	2.433	2.428
FNO	1.741	1.740	2.505	2.505	2.281
H_2_O	1.853	1.853	1.872	1.872	1.853
HCN	3.049	3.048	3.034	3.034	3.011
HNC	3.026	3.026	3.230	3.229	3.047

Table III reports the partial charges for C_2_H_4_, H_2_O, HCN, HNC and MgF_2_ calculated with B3LYP using the Mulliken, Löwdin, natural population analysis (NPA),^24,25^ and Bader’s AIM approaches. We note that with these methods, population analysis based on post-Hartree-Fock densities is either undefined or challenging to perform. Three different basis sets were used for each population analysis method. The MT B3LYP charges with the aug-cc-pVQZ basis set are also listed as a reference. It is well known that both the Mulliken and Löwdin analyses depend sensitively on the basis set. The AIM and NPA charges show good stability. The MT charges are most similar to the NPA charges. We note that neither the NPA nor AIM charges give correct dipole moments for the molecules except in trivial cases where the dipoles are zero due to symmetry. For the AIM approach, the difference in dipole is due to the first moment contribution from each atomic basin.

**TABLE III.** Comparison of partial charges determined by several population analysis methods for selected molecules. All charges were computed with 3 different basis sets using the B3LYP density except for the MT charges, where only the aug-cc-pVQZ results are reported. Much smaller dependence of MT charges on basis set (Table I) has been observed than any other population analysis methods. Only charges on symmetry-unique atoms are shown. AVXZ stands for the aug-cc-pVXZ basis set. All charges are reported in elementary charge units, *e*.

		C_2_H_4_		H_2_O		HCN			HNC			MgF_2_	
	Basis set	C	H	O	H	H	C	N	H	N	C	Mg	F
Mulliken	AVDZ	0.946	−0.473	−0.167	0.084	−0.093	0.380	−0.286	−0.004	0.207	−0.203	1.444	−0.722
	AVTZ	−0.621	0.311	−0.359	0.179	0.600	−0.325	−0.275	0.244	0.070	−0.314	1.309	−0.654
	AVQZ	0.369	−0.185	−0.582	0.291	0.550	−0.093	−0.457	0.268	−0.283	0.015	1.338	−0.669
Löwdin	AVDZ	−0.059	0.029	0.017	−0.008	0.038	−0.005	−0.033	0.050	−0.083	0.033	0.982	−0.491
	AVTZ	0.125	−0.063	0.399	−0.200	−0.064	0.003	0.061	−0.097	0.087	0.010	0.571	−0.286
	AVQZ	−0.150	0.075	0.875	−0.437	−0.184	0.025	0.159	−0.300	0.380	−0.080	0.295	−0.147
NPA	AVDZ	−0.413	0.207	−0.958	0.479	0.226	0.107	−0.333	0.455	−0.754	0.299	1.751	−0.875
	AVTZ	−0.372	0.186	−0.923	0.461	0.223	0.080	−0.303	0.436	−0.721	0.285	1.762	−0.881
	AVQZ	−0.386	0.193	−0.926	0.463	0.228	0.071	−0.299	0.444	−0.737	0.294	1.749	−0.875
AIM	AVDZ	0.002	−0.007	−1.166	0.583	0.214	1.024	−1.240	0.557	−1.574	1.015	1.788	−0.895
	AVTZ	0.023	−0.049	−1.138	0.569	0.209	0.893	−1.104	0.527	−1.479	0.951	1.778	−0.890
	AVQZ	−0.002	0.003	−1.135	0.567	0.188	0.963	−1.153	0.532	−1.504	0.971	1.772	−0.886
MT	AVQZ	−0.292	0.146	−0.655	0.327	0.259	0.050	−0.308	0.408	−0.220	−0.188	1.408	−0.704

**FIG. 2.**
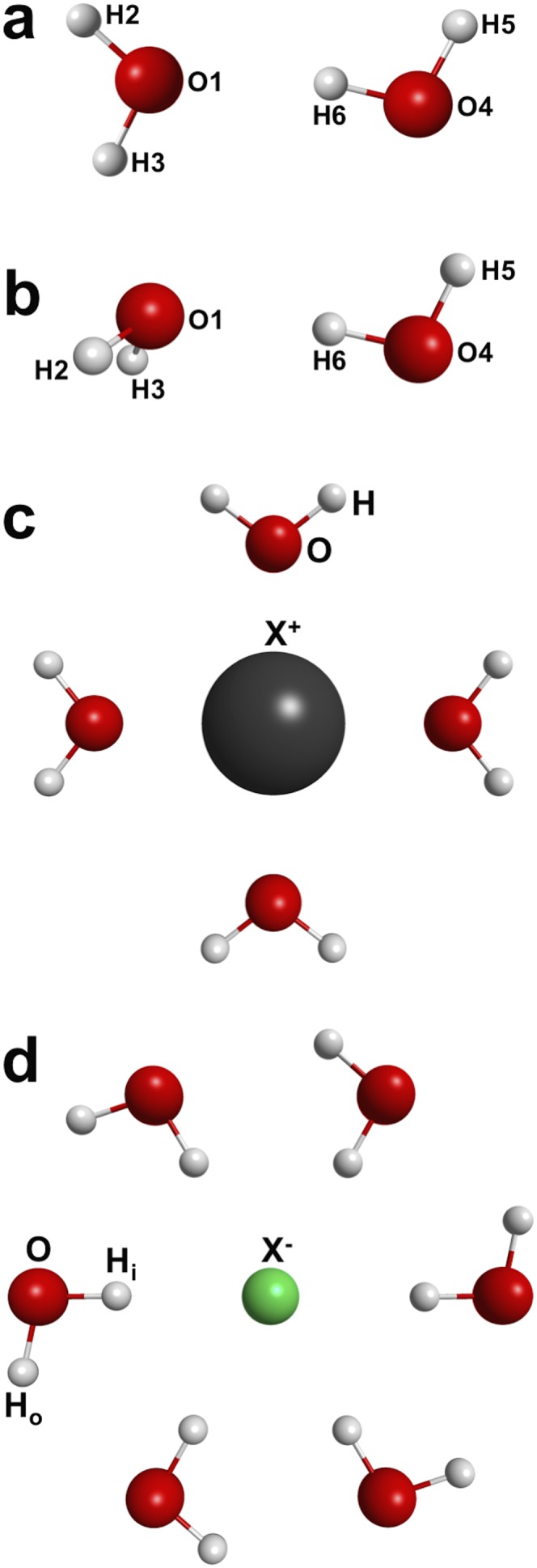
Geometry of the planar water dimer (a), geometry of the minimum energy water dimer (b), schematic diagram for the hydrated cation with D_nh_ symmetry (c), and schematic diagram for the hydrated anion with C_nh_ symmetry (d). Only symmetry unique atoms are labeled. The label used matches those listed in Table IV. The number of water molecules in the hydration shell is different for each ion, although only that corresponding to the number of water for Li^+^ and F^−^ are shown.

Table IV reports MT charges for a planar water dimer and a few hydrated ions. The global minimum for the water dimer [Fig. 2(b)] is also included, although in this case, only the MT charges on the proton donor water are reported. We note that for the non-planar water dimer, the charge flow term is not exactly zero as discussed previously. For the global minimum of the water dimer, however, the effect on the proton donor water is small. If the charges on the proton acceptor water atoms are measured using the local plane of the acceptor water, the contribution from the charge flow term will be slightly more significant. Since the main goal of this study is to gain a semi-quantitative understanding of the extent of the charge transfer in liquid water, we believe measuring the MT charge on the donor water using the symmetry plane of the dimer is sufficient. We note that one way to estimate the extent of charge flow contributions is to calculate the force in the reference plane as a result of the field perpendicular to the plane. While for a truly planar molecule, this force is very close to zero, indicating that the field is not changing the electron distribution to the first order; for non-planar molecules, this indicator of electron redistribution could become large.

**TABLE IV.** MT charges calculated for the planar (H_2_O)_2_, the global minimum of (H_2_O)_2_, planar hydrated cation, and planar hydrated anion systems. The CCSD(T) charges for the anionic systems are not available due to our electronic structure code failing some consistency tests. The name of the atoms can be found in Fig. 2. AVXZ stands for the aug-cc-pVXZ basis set; the use of corresponding core-valance basis set for the cations and the use of effective core pseudo-potentials for I^−^ are described in the text. All charges are reported in elementary charge units, *e*.

System	Atom	B3LYP AVQZ	MP2 AVQZ	CCSD(T) AVTZ
(H_2_O)_2_ planar	O1	−0.610	−0.613	−0.606
	H2	0.338	0.338	0.334
	H3	0.330	0.329	0.325
	O4	−0.650	−0.648	−0.642
	H5	0.319	0.320	0.316
	H6	0.274	0.274	0.274
(H_2_O)_2_ global minimum	O4	−0.656	−0.653	−0.648
	H5	0.316	0.317	0.314
	H6	0.263	0.263	0.262
Li^+^(H_2_O)_4_	Li^+^	0.609	0.617	0.620
	H	0.333	0.332	0.329
	O	−0.566	−0.568	−0.563
Na^+^(H_2_O)_4_	Na^+^	0.711	0.722	0.726
	H	0.330	0.330	0.327
	O	−0.587	−0.590	−0.584
K^+^(H_2_O)_4_	K^+^	0.764	0.771	0.776
	H	0.328	0.328	0.325
	O	−0.597	−0.598	−0.593
F^−^(H_2_O)_6_	F^−^	−0.477	−0.483	
	H_i_	0.203	0.244	
	O	−0.571	−0.567	
	H_o_	0.281	0.281	
Cl^−^(H_2_O)_7_	Cl^−^	−0.475	−0.479	
	H_i_	0.189	0.186	
	O	−0.544	−0.540	
	H_o_	0.279	0.279	
Br^−^(H_2_O)_8_	Br^−^	−0.494	−0.497	
	H_i_	0.189	0.185	
	O	−0.529	−0.524	
	H_o_	0.278	0.278	
I^−^(H_2_O)_8_	I^−^	−0.448	−0.447	
	H_i_	0.175	0.170	
	O	−0.519	−0.513	
	H_o_	0.275	0.274	

Similarly for the hydrated ion clusters, we will restrict our analysis to the planar hydration structures to completely remove charge flow contributions. Although such structures are hypothetical, we believe they are sufficient to provide a semi-quantitative estimate of the extent of the charge transfer between hydrated ions and liquid hydration water. This information could be important for force field developments.

For the planar hydration structures, the cluster size was selected so that the vertical hydration energy,

$$E_{hydration}=E_{ion-solvation}-E_{ion}-E_{solvation-shell},$$ (8)

is minimum for the number of water in the cluster. In this equation, *E*_*ion-solvation*_ is the total energy of the optimized ion-water cluster; *E*_*ion*_ is the energy of the ion, and *E*_*solvation-shell*_ is the energy of the empty solvation shell assuming the same geometry as in the ion-water cluster.

It has been argued that the proper treatment of core correlation is important for modeling the hydration of alkali ions.^26^ Thus the aug-cc-pCVQZ basis set^27^ was used for Li^+^, Na^+^, and K^+^ for the B3LYP and MP2 MT charge calculations. The water in the hydration shell was modeled with the aug-cc-pVQZ basis set for the determination of MT charges. For the MP2 and CCSD(T) calculations, the excitations from the *n-*1 shell electrons were included. Geometry optimizations were performed using the corresponding levels of theory with the aug-cc-pCVDZ/aug-cc-pVDZ basis sets.

For the water dimer and the cationic clusters, the MT charges were also calculated with CCSD(T) aug-cc-pVTZ and aug-cc-pCVTZ basis sets using the MP2 geometries. For anionic clusters, the CCSD(T) calculations fail our consistency test in that they do not satisfy the Millikan constraint, possibly indicating some problems with the electronic structure code we used. Thus, the anionic CCSD(T) MT charges were not reported. All calculations involving the iodide were performed with the aug-cc-pVQZ-PP basis set for I^−^ with the associated effective core pseudo-potential.^28^ Similar to the hydrated cations, all anionic MT charges were calculated with the aug-cc-pVQZ basis set with the geometries optimized with aug-cc-pVDZ.

For the planar water dimer configuration, with CCSD(T), our calculations indicate a small charge transfer of −0.052 *e* from the proton acceptor water to the proton donor water, with the proton acceptor being slightly positive. The MT charge measured on the 3D water dimer minimum also revealed a similar charge transfer of −0.072 *e*. The amount of the charge transfer predicted by MP2 and B3LYP is also similar. This is about an order of magnitude larger than the estimate of 0.0025 *e* based on the absolutely localized molecular orbital approach for the 3D water dimer minimum.^29^

The MT charges determined for the hydrated ions are much smaller than their valence charge of one in magnitude, indicating the possibility of an appreciable charge transfer between the ion and their hydration water molecules.

For cations, CCSD(T) shows an MT charge of 0.78 *e*, indicating possibly a 0.22 electron transfer from the hydration shell to the K^+^*,* whereas Li^+^ obtained almost 0.38 electron from the hydration waters. We note that this number may reflect a partial screening effect by the hydration water. The hydration water will have an induced dipole moment due to the external field. Although the induced dipole moment will not experience a force in the external field, it will weaken the electric field in the center of the ring. Thus the ion will not feel the total external field, similar to a ring current screening the magnetic field in NMR experiments. The induced dipoles on water will in turn feel a push due to the field gradients from the ionic charge, resulting in a redistribution of the forces. However, MT charges on the water are consistent with charge transfer being the leading reason for the smaller ionic charge. For the cationic ring, the oxygen has a less negative MT charge when compared to the −0.647 *e* charge in an isolated water. On the other hand, the hydrogen atoms have almost identical charges compared to the hydrogen atoms in an isolated water molecule. If the magnitude of in-plane induction is significant when compared to the charge transfer, one would anticipate the oxygen to be more negative due to induction. Since the in-plane induction is not significant, it is hard to imagine the induction due to the small external field to be significant considering the proximity of the ion to the water molecules. We note that for the hydrated anions, the water MT charges are also consistent with charge transfer to the lowest unoccupied molecular orbitals (LUMOs) of water (*vide infra*).

For anions, the amount of charge transfer is not monotonic from F^−^ to I^−^ according to both B3LYP and MP2. This trend is likely due to the interplay of several factors, such as the electronegativity, the size of the anion, and the number of water molecules in the hydration shells. With MP2, the hydrated F^−^, Cl^−^, and Br^−^ have a comparable charge transfer of 0.51 to 0.52 *e* from the ion to the hydration water. For I^−^, the amount of charge transfer increases to about 0.55 *e*. Thus overall, the anions have a stronger charge transfer than cations. Also, the MT charges on both hydrogen atoms are significantly less positive compared to the 0.324 *e* value in the isolated water monomer, consistent with charge transfer from the anion to the LUMO of water. The LUMO of water has more weight on the hydrogen atoms than on the oxygen.

Although the analysis of the charge transfer reviewed in Table IV shows appreciable charge transfer especially for the anions, we caution when a 2D system is studied as a proxy for the insight of charge transfer in a 3D system. However, we have no reason to believe that the extent of the charge transfer will be fundamentally different for hydrated ions in 3D. Other population analysis methods that give the best agreement with the MT charges for the same 2D systems might be the best techniques to investigate charge transfer in ionic solutions in 3D.

## III. SUMMARY AND CONCLUSION

By measuring the atomic forces of a planar molecule in a uniform, out-of-the-plane electric field, the MT atomic charges can be obtained. It has been shown that for a planar molecule, the electron density is not perturbed by any infinitesimal displacement of any atoms in the out-of-plane direction. Thus, the corresponding charge flow terms will be exactly zero. The procedure can be considered as an atomic version of the Millikan oil drop experiment.

The MT charge is directly proportional to the true atomic force experienced in a uniform external field; thus, it is an important quantity in the development of force fields. Unlike most population analysis methods, the determination of MT charges does not require direct knowledge of the electronic density, thus making it ideal for determining partial charges for correlated levels of theory. Determination of post-HF energies is significantly easier than solution of the corresponding wave-functions. In addition, the MT charges can be easily calculated with existing electronic structure programs as long as the program can evaluate the potential energy in the presence of an external field. The limitation of the atomistic version of the Millikan experiment is that the MT charges are free of charge-flow contributions only for a planar molecule. The reliability of similar approaches for 3-dimensional complexes is worth additional investigation.^15^

The MT charges for several planar molecules and molecular complexes were investigated at up to the CCSD(T) level of theory. The MT charges show good stability with respect to the use of different basis sets. The MT charges are most similar to the NPA charge for the few molecules studied in our work. However, the ease with which MT charges are obtained at post-HF levels is important, especially for challenging molecules such as FNO.

For a planar water dimer, the MT scheme establishes an intermolecular charge transfer of around −0.052 *e* from the proton acceptor water to the donor. This number is around −0.072 *e* for the water dimer global minimum. Whereas planar hydrated cations, K^+^, Na^+^, and Li^+^, show a moderate charge transfer of −0.22 *e* to −0.38 *e* from the waters to the ion, planar hydrated anions, F^−^, Cl^−^, Br^−^, and I^−^, show a more significant transfer of around −0.5 *e* from the ion to the waters*.* We do acknowledge there might be a minor reduction of the apparent MT charge at the center of the water hydration ring due to induced ring dipole screening the field on the ion.

## References

[1] R. S. Mulliken, J. Chem. Phys. 23(10), 1833–1840 (1955). 10.1063/1.1740588

[2] P.-O. Löwdin, J. Chem. Phys. 18(3), 365–375 (1950). 10.1063/1.1747632

[3] P.-O. Löwdin, Adv. Quantum Chem. 5, 185–199 (1970). 10.1016/S0065-3276(08)60339-1

[4] A. J. Stone, Chem. Phys. Lett. 83(2), 233–239 (1981). 10.1016/0009-2614(81)85452-8

[5] F. L. Hirshfeld, Theor. Chim. Acta 44(2), 129–138 (1977). 10.1007/bf00549096

[6] F. W. Bieglerkonig, T. T. Nguyendang, Y. Tal, R. F. W. Bader, and A. J. Duke, J. Phys. B: At., Mol. Opt. Phys. 14(16), 2739–2751 (1981). 10.1088/0022-3700/14/16/004

[7] R. Z. Khaliullin, A. T. Bell, and M. Head-Gordon, J. Chem. Phys. 128(18), 184112 (2008). 10.1063/1.2912041 18532804

[8] W. C. Lu, C. Z. Wang, M. W. Schmidt, L. Bytautas, K. M. Ho, and K. Ruedenberg, J. Chem. Phys. 120(6), 2629–2637 (2004). 10.1063/1.1638731 15268406

[9] G. Knizia, J. Chem. Theory Comput. 9(11), 4834–4843 (2013). 10.1021/ct400687b 26583402

[10] T. Janowski, J. Chem. Theory Comput. 10(8), 3085–3091 (2014). 10.1021/ct500245f 26588279

[11] R. A. Millikan, Philos. Mag. Ser. 6 19(110), 209–228 (1910). 10.1080/14786440208636796

[12] R. A. Millikan, Phys. Rev. 2(2), 109–143 (1913). 10.1103/physrev.2.109

[13] E. A. Davis and I. Falconer, J. J. Thomson and the Discovery of the Electron (Taylor & Francis, 1997).

[14] A. Milani and C. Castiglioni, J. Phys. Chem. A 114(1), 624–632 (2010). 10.1021/jp908146d 19888738

[15] A. Milani, M. Tommasini, and C. Castiglioni, Theor. Chem. Acc. 131(3), 17 (2012). 10.1007/s00214-012-1139-5

[16] M. Gussoni, C. Castiglioni, and G. Zerbi, J. Chem. Phys. 80(4), 1377–1381 (1984). 10.1063/1.446885

[17] J. Cioslowski, J. Am. Chem. Soc. 111(22), 8333–8336 (1989). 10.1021/ja00204a001

[18] A. Milani and C. Castiglioni, J. Mol. Struct.: THEOCHEM 955(1-3), 158–164 (2010). 10.1016/j.theochem.2010.06.011

[19] T. H. Dunning, J. Chem. Phys. 90(2), 1007–1023 (1989). 10.1063/1.456153

[20] D. E. Woon and T. H. Dunning, J. Chem. Phys. 98(2), 1358–1371 (1993). 10.1063/1.464303

[21] A. K. Wilson, D. E. Woon, K. A. Peterson, and T. H. Dunning, J. Chem. Phys. 110(16), 7667–7676 (1999). 10.1063/1.478678

[22] M. W. Schmidt, K. K. Baldridge, J. A. Boatz, S. T. Elbert, M. S. Gordon, J. H. Jensen, S. Koseki, N. Matsunaga, K. A. Nguyen, S. J. Su, T. L. Windus, M. Dupuis, and J. A. Montgomery, J. Comput. Chem. 14(11), 1347–1363 (1993). 10.1002/jcc.540141112

[c23] 23.Bug reports have been filed for various consistency issues experienced with GAMESS and other electronic structure software we used.

[24] A. E. Reed, R. B. Weinstock, and F. Weinhold, J. Chem. Phys. 83(2), 735–746 (1985). 10.1063/1.449486

[25] A. E. Reed, L. A. Curtiss, and F. Weinhold, Chem. Rev. 88(6), 899–926 (1988). 10.1021/cr00088a005

[26] J. C. Li and F. Wang, J. Phys. Chem. B 120(34), 9088–9096 (2016). 10.1021/acs.jpcb.6b06102 27464064

[27] D. Feller, E. D. Glendening, D. E. Woon, and M. W. Feyereisen, J. Chem. Phys. 103(9), 3526–3542 (1995). 10.1063/1.470237

[28] K. A. Peterson, D. Figgen, E. Goll, H. Stoll, and M. Dolg, J. Chem. Phys. 119(21), 11113–11123 (2003). 10.1063/1.1622924

[29] R. Z. Khaliullin, A. T. Bell, and M. Head-Gordon, Chem. - Eur. J. 15(4), 851–855 (2009). 10.1002/chem.200802107 19086050

